# Treatment of lipoid proteinosis due to the p.C220G mutation in ECM1, a major allele in Chinese patients

**DOI:** 10.1186/1479-5876-12-85

**Published:** 2014-04-04

**Authors:** Rong Zhang, Yang Liu, Yang Xue, Yinan Wang, Xinwen Wang, Songtao Shi, Tao Cai, Qintao Wang

**Affiliations:** 1 State Key Laboratory of Military Stomatology, Department of Periodontology, School of Stomatology, the Fourth Military Medical University, Xi’an 710032, P.R. China; 2 Department of Stomatology, The 309th Hospital of Chinese People’s Liberation Army, Beijing, P.R. China; 3 State Key Laboratory of Military Stomatology, Department of Oral Biology, School of Stomatology, the Fourth Military Medical University, Xi’an 710032, P.R. China; 4 Department of Plastic and Burns Surgery, Tangdu Hospital, the Fourth Military Medical University, Xi'an 710032, P.R. China; 5 Center for Craniofacial Molecular Biology, Herman Ostrow School of Dentistry, University of Southern California, Los Angeles, California, USA; 6 Oral Medicine Research Institute, School of Stomatology, the Fourth Military Medical University, Xi’an 710032, P.R. China

**Keywords:** Extracellular matrix protein 1, Lipoid proteinosis, Anaphylaxis, Glucocorticoid, Anaphylaxis, Treatment

## Abstract

**Background:**

Lipoid proteinosis (LP) is known to be resulted from mutations of the extracellular matrix protein 1 gene (*ECM1*). However, no effective or sustained therapeutic methods to alleviate LP symptoms have been reported.

**Methods:**

Here, we report a 12-year-old boy with LP and recurrent anaphylaxis. The laboratory and histopathological investigations were adopted to confirm the diagnosis, and gene sequencing was performed. We treated this patient with glucocorticoid for three years to relieve the patient’s lipid metabolism disorder and symptoms related to LP and anaphylaxis.

**Results:**

The Laboratory and histopathological investigations showed a lipid metabolism disorder and anaphylaxis in the patient. A homozygous missense mutation p.C220G of ECM1 was identified by Sanger sequencing, which is a major allele in Chinese patients with LP. Notably, after three years’ treatment, the symptoms such as skin lesions, stiff oral mucosa and hoarse voice in the patient were significantly relieved or recovered.

**Conclusions:**

Our report may provide a potentially effective therapeutic approach for the first time to other LP patients who are experiencing recurrent anaphylaxis and/or chronic inflammation.

## Background

Lipoid proteinosis (LP) (OMIM 247100), also known as Urbach-Wiethe disease, is a rare autosomal recessive genodermatosis characterized predominantly by hoarseness, variable scarring and infiltration of the skin and mucosa [1]. LP was first reported by Urbach and Wiethe in 1929, and originally named ‘lipoidosis cutis et mucosae’. This disorder typically presents warty skin infiltration, beaded papules along the eyelid margins, skin scarring, extracutaneous abnormalities, as well as hoarseness of the voice, epilepsy and neuropsychiatric abnormalities [2]. Histologically, there can be widespread deposition or accumulation of hyaline-like materials and disruption or irregular reduplication of basement membrane around blood vessels and at the dermal-epidermal junction. Since pathological mutations were identified in the extracellular matrix protein 1 gene (*ECM1*) in 2002, more than 50 different cases with *ECM1* mutations have been reported thus far, most of which were specific to individual families [3]. In this paper, we reported a homozygous mutation of *ECM1* gene in a Chinese boy with LP and recurrent anaphylaxis. Notably, we present our experience from a pilot study for treating the patient with therapeutic glucocorticoid.

## Methods

### Histopathological analysis

Informed consent was obtained from the patient’s parents. Biopsy specimens were taken from the patient’s thickened and stiff tongue mucosa. Normal mucosa as a control was obtained from surgical specimens. The specimens were fixed in 10% formalin and processed for routine light microscopy with paraffin embedding. Sections were stained with haematoxylin and eosin (HE) and periodic acid–Schiff (PAS).

### PCR and Sanger sequencing

Peripheral blood samples were taken from the affected patient and his parents. DNAs were extracted using Gentra Puregene DNA kit (Qiagen, Valencia, CA, USA). Primers were designed for amplification of all exons of the ECM1 gene (see Additional file 1). For PCR amplification, 250 ng of genomic DNA was used as the template in an amplification buffer containing 5 pmol of each primer, 2.5 mmol MgCl_2_, 0.5 mmol of each nucleoside triphosphate and 1.25 U of AmpliTaq Gold polymerase (Applied Biosystems, Foster City, CA, USA) in a total volume of 50 μl in a GeneAmp PCR System 9700 thermal cycler (Applied Biosystems, Foster City, CA). The amplification conditions were 95°C for 5 min, followed by 35 cycles of 95°C for 1 min, annealing temperature (see Additional file 1) for 45 s, 72°C for 45 s. PCR products were analyzed by 2.5% agarose gel electrophoresis and purified using QIAquick PCR Purification Kit (Qiagen, Valencia, CA, USA) for sequencing in an ABI 310 Genetic Analyzer (Applied Biosystems, Foster City, CA). The control samples were selected from 100 normal individuals.

### Clinical therapy

All the treatments were approved by the Ethics Committee of Stomatological Hospital of FMMU, PLA (IRB-REV-2013006). The patient was treated with 1 ml of compound betamethasone plus equivalent lidocaine by submucosal injection to the underlip and margo lateralis linguae monthly for a period of 6 months and were then administered every 2 months for another 6 months. After that, the patient was suggested to take hydrocortisone orally in the dosage of 20-25 mg per quadratmeter of body surface area and locally on the skin lesion every three days for 2 years. Clinical follow-up was carried out weekly for another one year to observe the endurance of the effect (see Additional file 2).

## Results

### Clinical manifestation

**Figure 1:**
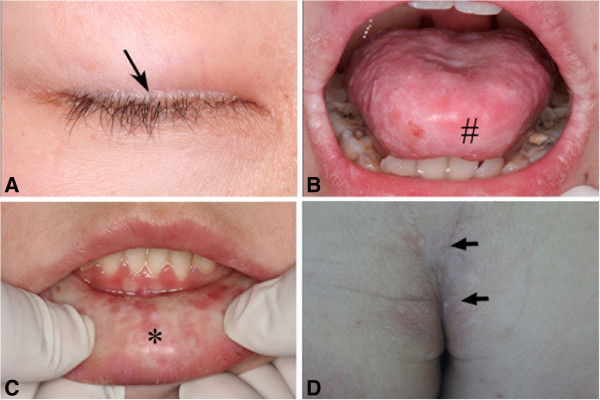
**Clinical features of lipoid proteinosis. A**. Beaded papules along the eyelids (indicated by an arrow); **B**. The patient’s tongue was hypertrophic and stiff; Movement of the tongue was restricted (#); **C**. The patient’s lower lip was also hypertrophic and stiff, with grainy materials (*); **D**. Waxy plaques and fine lines were shown on his buttock (by arrows).

**Figure 2:**
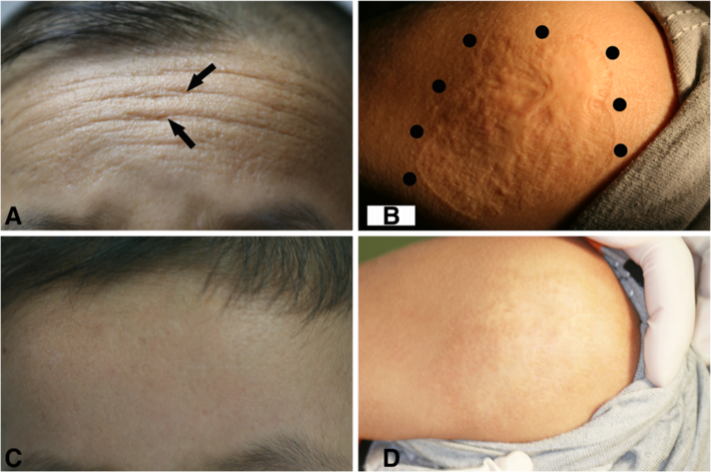
**Effects of the treatment. A**. Yellowish papular infiltration and fine lines on the patient’s forehead (indicated by arrows); **B**. Irregular and rugged scar on the skin of left shoulder (by dots); **C**. The popular and fine lines almost disappeared on the forehead; **D**. The scar on the left shoulder became flat, and the skin color also lightened.

A 12 year-old boy asked for management of sclerosis of oral mucosa in 2008. The patient was the only child of his nonconsanguineous parents. The patient had hoarseness since infancy, and experienced recurrent ulcerations on his oral mucosa and restricted tongue movement since he was three years old. From the age of 5 years, the patient had dry skin with numerous waxy plaques over his occipitalia, back, buttocks and antecubital fossa and vulnerable to minor trauma. After healing, the wound was easy to form scars. Moreover, his tongue and lips gradually became hypertrophic and stiff. Since then his parents found that the boy began to suffer from recurrent anaphylaxis to many kinds of anaphylactogen like pollen. Physical examinations revealed beaded eyelids papules (Figure 1A), hypertrophic lips and tongue with white and thicken mucosa (Figure 1B, C). His tongue was enlarged and restricted by a thickened frenulum. Waxy, yellow papules and nodules as well as deepening fine line were also noted on his buttocks and forehead (Figure 1D, Figure 2A). An irregular scar (about 7 × 7 cm^2^) was found on his left shoulder (Figure 2B). Neurological and psychological examinations were found to be normal.

### Laboratory and histopathological findings

**Figure 3:**
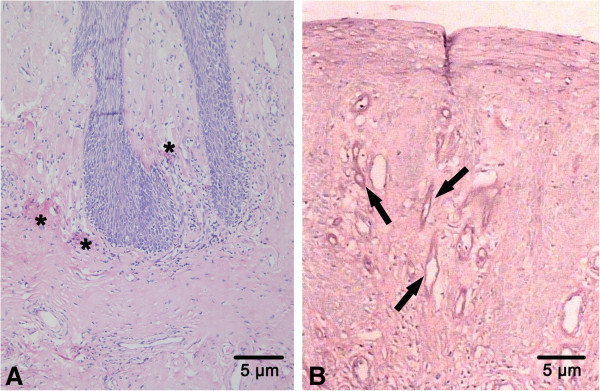
**Histological findings of lipoid proteinosis. A**. Hyperplasia of the mucous epithelium and deposits of homogeneous hyaline-like materials throughout the lamina propria in the patient’s tongue (*) (by haematoxylin and eosin staining); **B**. Hyaline-like materials surrounding several blood vessels (indicated by arrows) (by periodic acid–Schiff, i.e., PAS).

Blood and immunological evaluation revealed incremental levels of eosinophilic granulocytes, basophilic granulocytes and IgE. Erythrocyte sedimentation rate (ESR) was elevated. Lipid profile indicated a lipid metabolism disorder (Table 1). Acupuncture reaction test was positive. However, other types of immunoglobulin, C-reaction protein and autoantibodies were normal. A computerized brain scan (CT scan) and magnetic resonance imaging (MRI) had no abnormal findings. Video laryngoscopy revealed hypertrophic adenoid, swollen epiglottis and thicken vocal cords. Histologically, a slight hyperplasia of mucous epithelium and a widespread deposition of hyaline-like materials were found throughout the lamina propria, especially around blood vessels (Figure 3A). The materials were positive for periodic-acid–Schiff (PAS) stains (Figure 3B).

**Table 1:** Laboratory data before and after the three years clinical treatment

**Item**	**Before treatment**	**After treatment**	**Normal range**
Eosinophilic granulocyte	6.50	0.50	(0.02-0.52 ) × 10^9^/L
Basophilic granulocyte	3.30	0.08	(0-0.06) × 10^9^/L
IgE	371.62	79.74	(0-100) IU/mL
ESR_*_	58	12	(0-15) mm/h
TG_†_	1.88	1.08	(0.48-1.82) mmol/L
TC_‡_	5.15	4.43	(2.80-5.20) mmol/L
HDL_§_	0.71	1.66	(0.90-1.83) mmol/L
LDL_¶_	4.32	2.62	(0-3.12) mmol/L

_*_ESR, erythrocyte sedimentation rate; _†_TG, triglyceride; _‡_TC, total cholesterol; _§_HDL, high density lipoprotein; _¶_LDL, low density lipoprotein.

### Mutation analysis identified a major allele in Chinese patients with LP

**Figure 4:**
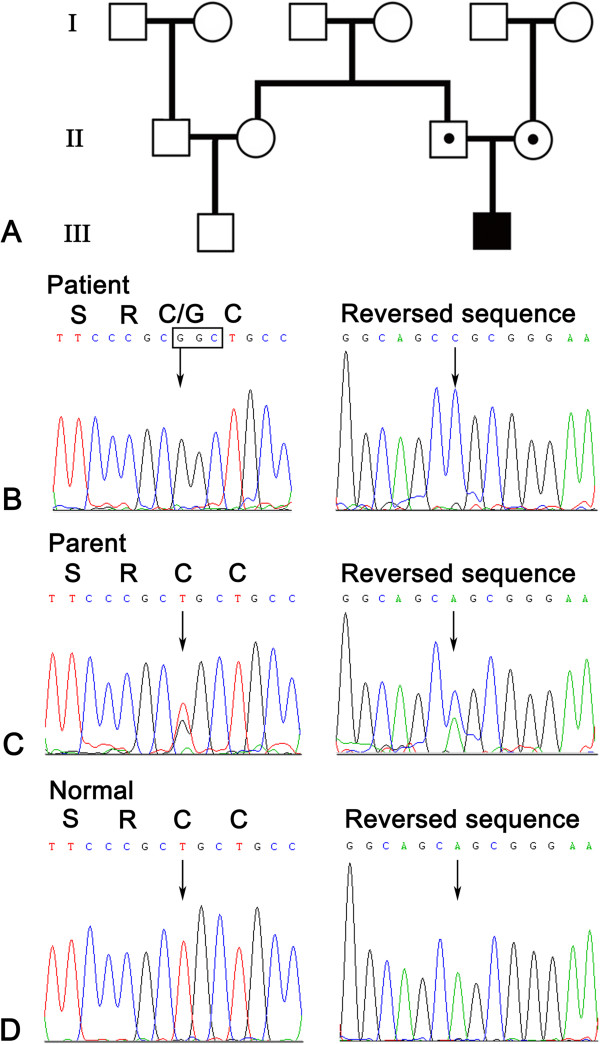
**Pedigree and sequencing result. A**. Pedigree of the family: the filled box represents the affected child, open box or circle with a dot, the heterozygote parents. **B**. Homozygous mutation c.658T > G (p.C220G) of ECM1 exon 6 of the patient. **C**. Heterozygous mutation of the same position of his parent. **D**. Normal DNA sequence of ECM1 exon 6.

Amplified DNA from the patient disclosed a homozygous T substitution to G at nucleotide 658 (c.658 T > G) in the exon 6 of the *ECM1* gene (Figure 4). This mutation converted cysteine to glycine, designated p.C220G. The parents were heterozygous for this mutation, which was not detected in 100 control genome DNAs (Figure 4A–D).

Literature analysis showed that in all reported eight Chinese patients from five unrelated families (Table 2), seven patients were found carrying the p.C220G mutation, accounting for 75% of disease allele. Five of them are homozygous and two are heterozygotes with compound mutations (p.C220G and p.R476X). Only one patient/family showed a different homozygous mutation (p.C450R). Also, the p.C220G mutation is predicted to be a damaging mutation by the Polyphen-2 program, suggesting it is a major rare causative mutation in Chinese patients. Furthermore, none of these three types of mutations have been found in the SNP database, Human Genome 1000 or in an additional 156 normal Chinese exome sequence database.

**Table 2:** Mutation analysis of Chinese patients with LP

**Patient number**	**Mutation position**	**Mutation type**	**Parents marriage**	**Hospital location**	**Ref.**
Two siblings	p.C220G	Homozygous	Unknown	Xi’an	Wang *et al*. [4]
Two siblings	p.C220G; p.R476X	Compound heterozygous	Non-consanguineous	Shanghai	Wang *et al*. [5]
Two siblings	p.C220G	Homozygous	Unknown	Shanxi	Han *et al*. [6]
One	p.C477R*	Homozygous	Non -consanguineous	Beijing	Liu *et al*. [7]
One	p.C220G	Homozygous	Non -consanguineous	Xi’an	This study
Total: 8	7 patients p.C220G				

Notes: Additional 18 cases were clinically described as LP, but specific ECM1 mutations were not determined [8]. *According to the updated reference sequence (GenBank acc. no., NP_004416.2), the mutation should be named as p.C450R.

### Clinical treatment

Based upon previous experience on treating lipid deposition diseases with glucocorticoids (see Additional file 3), we initiated a clinical treatment with a modified protocol for the patient (see Additional file 2). After 1 year of oral submucous injection and 2 years of oral and local application of glucocorticoid, the patient’s symptoms were significantly improved. His stiff underlip and lingual mucosa became softening; his hoarse voice returned to normal. The waxy, yellow papules and the deepening fine line on the forehead as well as the rugged scars on the left shoulder became flat, smooth and lightened (Figure 1C, D). No side effects were observed. Results of the hematological examination returned to the normal ranges except for a slightly higher basophilic granulocyte level (Table 1). Several other laboratory tests were also dramatically improved (see Additional files 4 &5).

## Conclusion

Lipoid proteinosis is characterized by various degrees of scarring and infiltration of skin and mucosae [1,9]. The typical clinical features include hoarseness, beaded eyelid papules, mucosae infiltration of the pharynx, tongue, soft palate, tonsils and lips [10,11]. In addition, the fragile skin may be easily damaged by minor trauma or friction, resulting in blisters and scar formation. All of these could be found in our case. Furthermore, histopathological findings of periodic acid–Schiff (PAS)-positive, and deposition or accumulation of hyaline materials in the lamina propria, as well as irregular hyperplasia of epithelium also strongly supported our diagnosis. To date, at least 47 different mutations in the *ECM1* gene have been reported for more than 50 unrelated patients with LP [3]. Most of them were family specific except for the largest groups of LP patients worldwide in Namaqualand, South Africa, suggesting a founder effect [12]. Approximately half of all mutations (22 of 47) were located within exon 6 or 7, suggesting a hot spot of mutations for this disorder. In this study, we identified a homozygous mutation also located on the exon 6 (c.658 T > G), which was previously observed in three additional unrelated Chinese families [4]. Since this mutation has not been detected in patients from any other races, it may represent an ancestral allele in Chinese Han population.

The human *ECM1* gene was isolated in 1997 and mapped to chromosome 1q21 [13]. ECM1 can stimulate blood vessel endothelial cell proliferation and angiogenesis. Within the epidermis, however, ECM1 is able to influence the differentiation of keratinocyte. After secreted into the dermis, ECM1 acts as a “biological glue” by binding to glycosaminoglycans and fibrillar protein growth factors, and then regulates basement membrane and interstitial collagen fibril macro-assembly and growth factor binding. Therefore, a loss-of-function mutation in *ECM1* gene may induce a strange pattern of keratinocyte maturation and differentiation, as well as dysregulation of dermal homeostasis and clinical features of skin infiltration and scarring [12,14] (see Additional file 6).

Although many therapeutic trials have been tested to alleviate LP symptoms, including oral steroids, oral dimethyl sulphoxide (DMSO) and intralesional heparin, as well as D-penicillamine and acitretin [9,15-17], no convincing evidence has been found to support any sustained treatment benefits. In our study, local injection of compound betamethasone and oral application of hydrocortisone have dramatically alleviated the patient’s symptoms such as thickened mucosa and recurrent anaphylaxis, and the treatment was well tolerated. We postulate that one of the possible mechanisms underlying it might be associated with the inhibitory effects of glucocorticoid on the matrix metalloproteinases (MMP-9) functions. Firstly, anaphylaxis with elevated IgE may activate mast cell to secrete tumor necrosis factor alpha (TNF-α) and to induce the proMMP-9 to be an active enzyme [18]. Secondly, the activated mast cell will further induce the MMP-9 to be released from fibroblasts through both adhesive interactions and the release of TNF-α from mast cells itself [19,20]. Thirdly, MMP-9 activation and overproduction are proved to be associated with the occurrence and development of some inflammatory reaction and anaphylaxis [21,22]. We thus assume that the application of glucocorticoid, by targeting the MMP-9 molecule, a key mediator in both LP and anaphylaxis, as well as in some inflammations, would alleviate the anaphylactic reaction in skin and mucosa lesions in LP. The postulated mechanism underling the effect of glucocorticoid on LP patients is shown (see Additional file 6). However, further experiments and prospective, randomized, controlled clinical trials are in need to verify this hypothesis and long-term therapeutic effects as well as the safety of glucocorticoid for treatment of LP.

In summary, we identified a hot C220G mutation of the *ECM1* gene in a child with LP, suggesting a founder effect for this allele in Chinese patients. More importantly, modified glucocorticoid application can significantly improve the symptoms of the patient suffering from LP and recurrent anaphylaxis with no side effects. Our experience and therapeutic protocol could be applied and verified in appropriate LP patients particularly complicated with recurrent anaphylaxis or associated chronic inflammation.

## Abbreviations

LP: Lipoid proteinosis; ECM1: Extracellular matrix protein 1; HE: Haematoxylin and eosin; PAS: Periodic acid–Schiff; PCR: Polymerase chain reaction; ESR: Erythrocyte sedimentation rate; MMP-9: Matrix metalloproteinases 9; TNF-α: Tumor necrosis factor.

## Competing interest

As a disclaimer, Tao Cai represented his own perspective in the paper, not the NIDCR/NIH. All remaining authors declare the absence of any Conflict of Interest.

## Authors’ contributions

RZ and YL performed mutation analysis and data interpretation, drafted the manuscript; YX, performed data analysis; YW, performed quality control of pathological data; RZ, YL and XW participated in samples’ collection and data acquisition; SS, performed pathological review and data analysis, participated into the design of the study; TC and QW performed data interpretation, conceived of the study, helped to draft the manuscript. All authors read and approved the final manuscript.

## Supplementary Material

Additional file 1 Primers used for PCR amplification of the ECM1 gene. Click here for file

Additional file 2 Protocol of Treatment for the patient with LP. Click here for file

Additional file 3 Lipid deposition disease treated with glucocorticoids. Click here for file

Additional file 4 Blood cell analysis before and after the three years clinical treatment. Click here for file

Additional file 5 Immunoglobulins, autoantibodies and T lymphocyte subsets in blood serum before and after the three years clinical treatment. Click here for file

Additional file 6 **A postulated mechanism of the treatment by glucocorticoid.** IgE, immunoglobulin E; TNF-α, tumor necrosis factor-α; MMP-9, matrix metalloproteinase 9; ECM1, extracellular matrix protein. Click here for file
