# Supplementary material for: Treatment of lipoid proteinosis due to the p.C220G mutation in ECM1, a major allele in Chinese patients
Source: J Transl Med. 2014 Apr 4;12:85. doi: 10.1186/1479-5876-12-85 (PMC4021827; doi:10.1186/1479-5876-12-85)
Supplement: Additional file 1 — Primers used for PCR amplification of the ECM1 gene. [file 1479-5876-12-85-S1.doc]

**Additional file 1. Primers used for PCR amplification of the ECM1 gene**

| Exon no | Forward primer (5’-3’) | Reverse primer (5’-3’) | Annealing temp. (℃) | Product size (bp) |
| --- | --- | --- | --- | --- |
| 1 | agctgggactgagtcatggc | taaaggctccactggcctag | 62 | 416 |
| 2/3 | tcctacactcttgatctcca | agaaacctggagggtcactg | 58 | 247 |
| 4/5 | cagtgaccctccaggtttct | cagagcccaccgtcttgtct | 56 | 484 |
| 6 | agccttgagaagcaggagga | agtgaacgggacctgaggtt | 54 | 671 |
| 7 | aacctcaggtcccgttcact | acatggatggatggactggc | 52 | 548 |
| 8 | cacatcaacagttgcctcct | ggcatcttctggcatcagat | 60 | 499 |
| 9 | agttgcctagtccttcccca | aggccaggtcagagtgaaga | 60 | 408 |
| 10 | aatccagctgtgcaaggcag | gtaatgagtgttcagatggg | 62 | 469 |
