# Supplementary material for: Treatment of lipoid proteinosis due to the p.C220G mutation in ECM1, a major allele in Chinese patients
Source: J Transl Med. 2014 Apr 4;12:85. doi: 10.1186/1479-5876-12-85 (PMC4021827; doi:10.1186/1479-5876-12-85)
Supplement: Additional file 2 — Protocol of Treatment for the patient with LP. [file 1479-5876-12-85-S2.doc]

**Additional file 2. Protocol of** Treatment for the patient with LP

| Step | Medicine | Method | Location | Duration | Effect |
| --- | --- | --- | --- | --- | --- |
| 1 | Betamethasone plus equivalent lidocaine | 0.1％ in 2 ml, submucosa injection | underlip and margo lateralis linguae | monthly for 6 months | stiff underlip and lingual mucosa became soften |
| 2 | the same as above | the same as above | the same as above | bimonthly for 6 months | hoarseness was improved |
| 3 | Hydrocortisone | 20-25 mg per quadratmeter of body surface area, oral and local application | orally and locally on the skin lesion | every three days for 2 years | waxy, yellow papules and the deepening fine line on the forehead as well as the rugged scars on the left shoulder  became flat and smooth |
| 4 | No | follow up | the same as above | monthly for 1 year | No recurrent symptoms |
