# Supplementary material for: Treatment of lipoid proteinosis due to the p.C220G mutation in ECM1, a major allele in Chinese patients
Source: J Transl Med. 2014 Apr 4;12:85. doi: 10.1186/1479-5876-12-85 (PMC4021827; doi:10.1186/1479-5876-12-85)
Supplement: Additional file 3 — Lipid deposition disease treated with glucocorticoids. [file 1479-5876-12-85-S3.doc]

**Additional file 3. Lipid deposition disease treated with glucocorticoids**

| Disease | Pathogeny | Clinical manifestations | Pathologic features | Treatment | Effects | Mechanism | Reference |
| --- | --- | --- | --- | --- | --- | --- | --- |
| Lipid Storage Myopathy | Defects in intracellular triglyceride (TG) catabolism, transport of long-chain fatty acids and carnitine, or fatty acid β-oxidation | Progressive muscle weakness | Lipid accumulation (muscle) | prednisone（12.5-60） mg/d for 4.5 months | The lipid material decreased markedly and strength was regained | Augmentation of the lipolytic effects, or a direct activating effect on triglyceride lipase | Andrew G et al. |
| Gaucher disease accompanied by antiphospholipid syndrome | Deficiencies of the lysosomal hydrolase glucocerebrosidase;  Antiphospholipid antibody | Hepatosplenomegaly, bone pain, hemolysis and spontaneous abortions | Glucosylceramide accumulation (macrophages);  Elevated levels of IgG and IgM anticardiolipin antibodies | prednisone (7.5-10) mg/d, aspirin  100 mg/day and imiglucerase replacement therapy for about 11 months | Became almost asymptomatic  and had successful pregnancy | Not mentioned | Y. Sherer et al. |
| Fabry’s Disease | Deficiencies of α-galactosidase A | Sudden visual loss secondary to ischemic optic neuropathy and characteristic whorl-like corneal deposits in both eyes in order of priority | Accumulation of glycosphingolipid in the endothelial and epithelial cells of the glomerulus and distal tubules | A starting dose of (60-80) mg prednisolone, which was slowly tapered thereafter, and 60,000 IU/d urokinase given intravenously for 1.5 months | The visual acuity and the visual field had improved, the optic disk edema and the cotton wool patch disappeared | Not mentioned | Abe H et al. |
| Juvenile neuronal ceroid Lipofuscinosis | An immune-mediated reaction to GAD65 | Mental decline and motor system weakness | Positive GAD65-antibodies in serum | Prednisolone 0.75 mg/kg, maximum dose of 40 mg,  per day orally for 10 consecutive days each month for 1 year | GAD65-antibodies turned negative and a motor and verbal IQ improvement was found | Not mentioned | Aberg L et al. |
| Cholesterol Ester Storage Disease | Lysosomal acid lipase deficiency | Hepatosplenomegaly,hyperlipidemia, bilateral adrenal calcificationsand disabling neurological manifestations | Lipid deposition in bloated macrophages of bone marrow | low-fat diet and oral  prednisolone (dose unknown) | The general conditions were improved significantly | Not mentioned | Bindu PS et al. |
