# Supplementary material for: Treatment of lipoid proteinosis due to the p.C220G mutation in ECM1, a major allele in Chinese patients
Source: J Transl Med. 2014 Apr 4;12:85. doi: 10.1186/1479-5876-12-85 (PMC4021827; doi:10.1186/1479-5876-12-85)
Supplement: Additional file 4 — Blood cell analysis before and after the three years clinical treatment. [file 1479-5876-12-85-S4.doc]

**Additional file 4. Blood cell analysis before and after the three years clinical treatment**

| Item | Before treatment | After treatment | Normal Range |
| --- | --- | --- | --- |
| white blood cell | 6.6 | 6.7 | (4.0-10.0) ×10^9^/L |
| lymphocyte | 3.3 | 2.4 | (0.8-4.0) ×10^9^/L |
| neutrophile granulocyte | 2.7 | 3.8 | (2.0-7.0) ×10^9^/L |
| monocyte | 0.6 | 0.3 | (0.1-0.9) ×10^9^/L |
| Lymphocyte％ | 49.8 | 35.9 | (20.0-50.0) ％ |
| neutrophile granulocyte％ | 41.4 | 56.1 | (40.0-75.0) ％ |
| monocyte％ | 8.8 | 4.9 | (3.0-9.0) ％ |
| red blood cell | 3.7 | 4.1 | (4.3-5.8) ×10^12^/L |
| hemoglobin | 110 | 119 | (130-175) g/L |
| [mean corpuscular volume](http://www.iciba.com/mean_corpuscular_volume) | 78.5 | 89.9 | (82.0-95.0) fL |
| mean corpuscular hemoglobin | 29.5 | 29.3 | (27.0-31.0) pg |
| mean corpuscular hemoglobin concentration | 376 | 326 | (320-360) g/L |
| platelet | 196 | 139 | (100-300) ×10^9^/L |
| platelet distribution width | 16.5 | 23.1 | (12-18) fL |
| mean platelets  volume | 11.1 | 14.5 | (4.0-12.0) fL |
