# Supplementary material for: Treatment of lipoid proteinosis due to the p.C220G mutation in ECM1, a major allele in Chinese patients
Source: J Transl Med. 2014 Apr 4;12:85. doi: 10.1186/1479-5876-12-85 (PMC4021827; doi:10.1186/1479-5876-12-85)
Supplement: Additional file 5 — Immunoglobulins, autoantibodies and T lymphocyte subsets in blood serum before and after the three years clinical treatment. [file 1479-5876-12-85-S5.doc]

**Additional file 5. Immunoglobulins, autoantibodies and T lymphocyte subsets in blood serum before and after the three years clinical treatment**

| Inspection Item | Before treatment | After treatment | Normal Range |
| --- | --- | --- | --- |
| IgG | 1440.0 | 1370.0 | (751.0-1560.0) mg/dl |
| IgA | 193.0 | 253.0 | (82.0-453.0) mg/dl |
| IgM | 188.0 | 218.0 | (40.0-274.0) mg/dl |
| alexin C3 | 120.0 | 111.0 | (79.0-152.0) mg/dl |
| alexin C4 | 18.0 | 21.0 | (16.0-38.0) mg/dl |
| antinuclear  antibodies | W+ | - | - |
| anti-dsDNA | W+ | - | - |
| anti-SSA | - | - | - |
| anti-SSB | - | - | - |
| anti-SCL-70 | - | - | - |
| anti-S-M | - | - | - |
| anti-Cenp-B | - | - | - |
| anti-Histones | W+ | - | - |
| anti-Jo-1 | - | - | - |
| anti-U1-nRNP | - | - | - |
| anti-rib | - | - | - |
| anti-RO-52 | - | - | - |
| anti-nucleosome | W+ | - | - |
| CD3+ CD4+％ | 43.0 | 49.0 | (34.0-70.0) ％ |
| CD3+ CD8+％ | 40.0 | 36.0 | (25.0-54.0) ％ |
| Th/Ts | 1.08 | 1.37 | (0.68-2.47) |
