# Supplementary material for: Treatment of lipoid proteinosis due to the p.C220G mutation in ECM1, a major allele in Chinese patients
Source: J Transl Med. 2014 Apr 4;12:85. doi: 10.1186/1479-5876-12-85 (PMC4021827; doi:10.1186/1479-5876-12-85)
Supplement: Additional file 6 — A postulated mechanism of the treatment by glucocorticoid. IgE, immunoglobulin E; TNF-α, tumor necrosis factor-α; MMP-9, matrix metalloproteinase 9; ECM1, extracellular matrix protein. [file 1479-5876-12-85-S6.doc]

**Additional file 6. A postulated mechanism of the treatment by glucocorticoid.**

IgE, immunoglobulin E; TNF-α, tumor necrosis factor-α; MMP-9, matrix metalloproteinase 9; ECM1, extracellular matrix protein
